# In Vitro Toxicokinetics and Phase I Biotransformation of the Mycotoxin Penitrem A in Dogs

**DOI:** 10.3390/toxins12050293

**Published:** 2020-05-04

**Authors:** Silvio Uhlig, Lada Ivanova, Pauline Voorspoels, Christiane Kruse Fæste

**Affiliations:** 1 Toxinology Research Group, Norwegian Veterinary Institute, Ullevålsveien 68, 0454 Oslo, Norway; lada.ivanova@vetinst.no (L.I.); christiane.faste@vetinst.no (C.K.F.); 2 Department of Bioanalysis, Faculty of Pharmaceutical Sciences, Ghent University, Ottergemsesteenweg 460, 9000 Gent, Belgium; pauline.voorspoels@gmail.com

**Keywords:** dogs, in vitro, liver microsomes, metabolite identification, penitrem A, poisoning, toxicokinetics

## Abstract

The tremorgenic mycotoxin penitrem A is produced by *Penicillium* species as a secondary metabolite on moldy food and feed. Dogs are sometimes exposed to penitrem A by consumption of spoiled food waste or fallen fruit. The lipophilic toxin crosses the blood-brain barrier and targets neuroreceptors and neurotransmitter release mechanisms in the central and peripheral nervous systems. Typical symptoms of penitrem A intoxication are periodical or continuous tremors, which can be passing, persistent or lethal, depending on the absorbed dose. There is presently no information on the biotransformation and toxicokinetics of penitrem A in dogs. The aim of the present study was therefore to identify potential metabolites of the toxin by performing in vitro biotransformation assays in dog liver microsomes. Analyses by liquid chromatography coupled to high-resolution mass spectrometry led to the provisional identification of eleven penitrem A phase I metabolites, which were tentatively characterized as various oxidation products. Furthermore, elimination parameters determined in in vitro assays run under linear kinetics were used for in vitro-to-in vivo extrapolation of the toxicokinetic data, predicting a maximal bioavailability of more than 50%. The metabolite profile detected in the in vitro assays was similar to that observed in the plasma of an intoxicated dog, confirming the predictive capability of the in vitro approach.

## 1. Introduction

**Figure 1:**
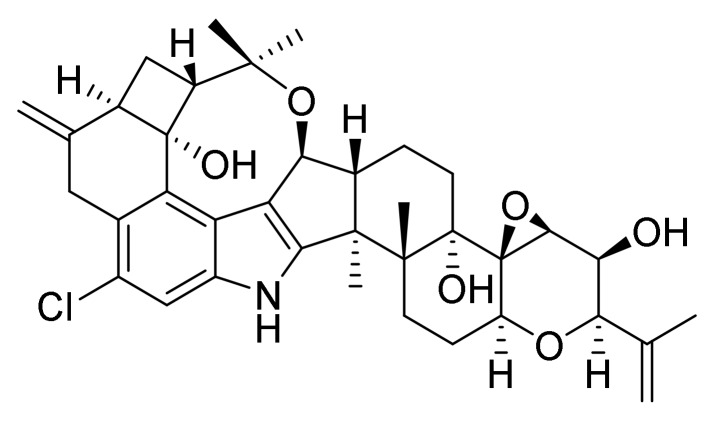
Chemical structure of the fungal neurotoxin penitrem A.

Penitrem A (Figure 1) is a toxic secondary metabolite of *Penicillium crustosum*, a fungus adapted to all climate zones that occurs mainly on spoiled food and feed [1,2]. It has, however, also been found on *Poa huecu* grass and in soil samples [3,4]. Intoxication with penitrem A from the ingestion of moldy foodstuffs or contaminated grass causes neurological symptoms, in which the severity and persistence depend directly on the level of exposure [5]. The mycotoxin can pass through the blood-brain barrier into the central nervous system, where it affects the GABAergic neurotransmission, blocks potassium channels and causes cell death [6,7]. Neurological effects such as activity suppression, lethargy and cataleptic behavior can occur as early as 30 s after uptake, followed by irritability, hyperresponsiveness to external stimuli (e.g., touch and noise) and muscle tremors, weakness and rigidity, which can proceed further to opisthotonus, mydriasis, nystagmus, convulsions and ataxia, and ultimately death [8,9,10].

Cases of suspected penitrem A-induced human mycotoxicosis are rare. The few known incidents are connected to the consumption of spoiled beer, food or inhalation from moldy silage [9,11]. “Huecu’s disease”, a ryegrass stagger-like syndrome, has been observed in Argentina in grazing animals including sheep, horses, cattle and goats [5]. Penitrem A intoxication affects, however, most frequently dogs due to their easy access to food waste and moldy fruit [11,12,13,14,15,16,17,18,19,20,21]. In addition to the neurological symptoms, penitrem A toxicosis in dogs is typically also accompanied by excessive salivation, vomiting, frequent urination and defecation, panting, metabolic acidosis and hyperthermia [1,10,13,22]. Depending on the level of toxin exposure, dogs recover after weeks or maybe months, but in severe cases, the coordination of muscle movements may still be impaired even after years [20]. As antidotes are not available, only alleviating care is possible, for example, by administration of activated charcoal, diazepam or barbiturates [10,23]. The marine drugs astaxanthin and docosahexaenoic acid have shown some effectiveness in counter-acting penitrem A-related cytotoxicity when applied preventively, but a curative treatment after intoxication has not been developed [24].

Penitrem A is a decacyclic indole–diterpenoid derived from the biosynthetic intermediate paspaline in a multi-step reaction pathway (Figure 1). The penitrem A molecule contains a fused cyclohexyl-cyclobutyl ring that is formed by two cyclized isoprene units attached to a tryptophan-derived indole moiety [6,9]. Functional groups include hydroxyl groups, chlorine and an epoxide ring.

Analytical methods for the selective determination of penitrem A in cell cultures, serum, urine, vomit, liver, brain, intestine and different foodstuffs most often make use of high performance liquid chromatography–tandem mass spectrometry (HPLC-MS/MS) [16,25,26,27,28,29]. After extraction from the matrix and clean-up, the toxin was separated by reversed-phase LC, ionized by positive electrospray ionization (ESI) or atmospheric pressure chemical ionization (APCI), fragmented by collision-induced dissociation (CID) and detected by triple quadrupole or linear ion trap MS/MS. Detection limits ranging from 0.7 µg/kg in cheese, 1 µg/kg in serum and urine to 5 µg/kg in a food mixture and different organs have been achieved. The penitrem A molecule loses water during ionization and produces characteristic product ions in MS/MS analyses that are used for unambiguous identification [27].

The lipophilic toxin is rapidly absorbed from the gastrointestinal tract after the ingestion of contaminated foodstuff [9]. Taking the onset of the neurological effects as an indicator, maximum plasma concentrations are probably reached within 30 min in mice, rats, dogs, sheep, pigs and cows. Penitrem A is quickly distributed to most body compartments, reaching its main site of action in the brain [28]. Biotransformation of the toxin has been studied in vivo in mice and in vitro with rat liver microsomes and primary hepatocytes [27], showing the formation of oxidized metabolites with increased hydrophilicity. Conjugation products were not observed, although excretion via bile and feces has been suggested as the main route of excretion [21].

Although intoxication of dogs is by far the most frequent adverse incident connected to penitrem A, information on the toxin’s biotransformation and toxicokinetics in dogs is presently not available. It was therefore the aim of the present study to determine potential products of the canine penitrem A metabolism and use in vitro-to-in vivo extrapolation (IVIVE) to predict main kinetic parameters [30].

## 2. Results and Discussion

### 2.1. Mass Spectrometric Fragmentation of Penitrem A

**Figure 2:**
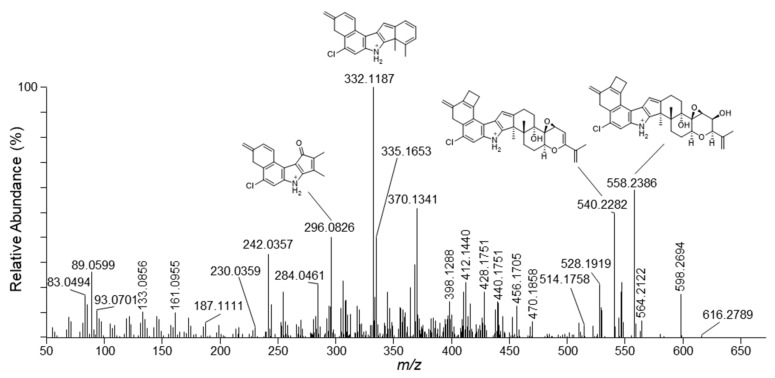
Product ion spectrum from higher-energy collision dissociation (HCD) of the [M+H]^+^ ions of penitrem A (^35^Cl isotopologue) including plausible structures of major fragment ions.

The mass spectrometric fragmentation of penitrem A (C_37_H_44_NO_6_Cl; exact mass of protonated molecules 634.2930 *m*/*z*) has been studied previously using collision-induced dissociation (CID) in a low-resolution ion-trap instrument [27]. CID resulted in sequential losses of water and acetone, and the mass spectra did not show any ions with significant signal intensities below *m*/*z* 500. Higher-energy collision dissociation (HCD) is a complementary technique that often results in more complex fragmentation, which may yield more structural information. We used HCD in a high-resolution tandem mass spectrometer (HRMS/MS) resulting in product ions over the entire mass range (Figure 2). While the principal product ion from CID was observed at *m*/*z* 558 [27], this ion was of medium intensity in the HCD HRMS/MS spectrum (Figure 2). However, the high-resolution approach verified that the product ion at *m*/*z* 558 was the result of sequential loss of acetone and water (−C_3_H_8_O_2_, i.e., –(C_3_H_6_O+H_2_O)) (Table 1). Furthermore, the transition from *m*/*z* 634 to *m*/*z* 558 has been used for the LC–MS/MS based quantitative penitrem A analyses in different matrixes, while *m*/*z* 616 and *m*/*z* 540 were detected as qualifier ions [26,29].

HCD generated several product ions that were the result of cleavage across the cyclic backbone of penitrem A. Examples for conceivable structures of the corresponding product ions are shown in Figure 2. The rationale behind studying the HRMS/MS spectrum of penitrem A was to apply the acquired data for the subsequent extraction of putative biotransformation products from all-ion-fragmentation (AIF) chromatograms.

### 2.2. Analysis of Penitrem A Metabolites Produced by In Vitro Biotransformation

**Figure 3:**
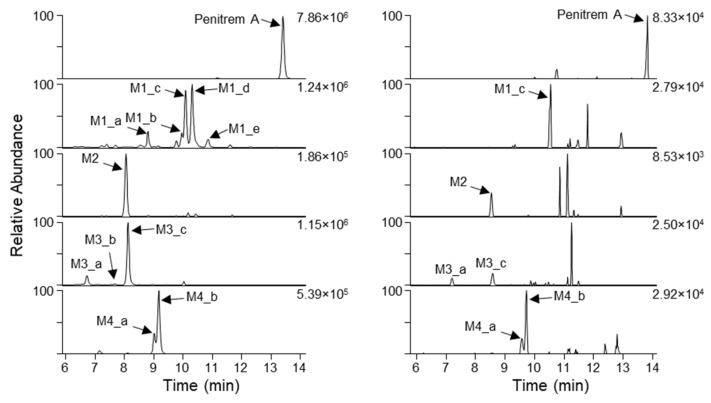
Extracted ion LC–HRMS chromatograms (±3.0 ppm) for [M+H]^+^ of penitrem A and putative phase I metabolites produced by incubation with dog liver microsomes (60 min, left panel) and in a plasma sample from an intoxicated dog (right panel). The shift to slightly longer retention times in the case of the plasma sample was due to the use of a different UHPLC mixer volume. The intensities of the highest peak in each chromatogram are indicated in the upper right-hand corners (arbitrary units).

Potential penitrem A metabolites produced by dog liver microsomes were characterized based on the detailed analysis of the FullMS data for different incubation time points and AIF chromatograms, using the specific MS/MS product ions of penitrem A for comparison. This resulted in the tentative assignment of eleven penitrem A biotransformation products (Figure 3, Table 2). Their elemental composition differed from that of penitrem A by addition of one or two oxygen atoms (M1_a–M1_e and M3_a–M3_c, respectively), formal addition of water (M2) or a combination of both (M4_a and M4_b) (Figure 3, Table 2). Isomeric metabolites with identical molecular formulae but different retention times (t_R_) were designated with identical metabolite numbers but with consecutive alphabetical suffixes. The main metabolites after incubation for one hour were mono-oxygenated (*m*/*z* 650.2858–650.2876) and mono-oxygenated/hydrated species (*m*/*z* 668.2984–668.2987).

Previously, up to five different hepatic penitrem A metabolites were observed either in vitro in incubations with rat liver microsomes or primary rat hepatocytes, or in vivo in mice after oral administration [27,28]. The samples were analyzed by low-resolution MS that did not allow the calculation of elemental compositions. However, the mass differences between penitrem A and the observed putative hepatic metabolites in the rodent experiments were comparable to those observed for the dog liver microsomes in the present study, except for a putative oxidation/dehydration product that was only detected in the livers of exposed mice and observed with a mass difference of −2 *m*/*z* relative to penitrem A. The increased number of identified metabolites in our study could also arise from a notable difference in the chromatographic analysis. While the previously reported HPLC methods used more traditional separation columns that probably did not have the ability to separate different isomers, we used a UHPLC method that was optimized for improved chromatographic resolution (longer column, smaller particles, core-shell instead of fully porous material). It could therefore be possible that penitrem A was also metabolized into isomeric metabolites in the rodent models, but that the analytical methodologies were insufficient to determine these.

### 2.3. Detection of Penitrem A and Metabolites in Intoxicated Dogs

In the course of the present study, we received samples from veterinary clinics of four dogs that showed signs of potential penitrem poisoning. It is unknown how much time had passed between the actual poisoning incident and the collection of samples. The samples consisted of either plasma or serum, and in addition, the stomach content of one dog. UHPLC–HRMS analyses showed that the stomach content of this dog was positive for penitrem A (data not shown). Six of the 11 penitrem A metabolites found in the dog liver microsomes could be confirmed in the plasma sample, while the stomach contents did not contain detectable amounts of metabolites, which is expected in this pre-systemic sample (Figure 3). Although signal intensities were too low for the acquisition of HRMS/MS spectra, we were able to tentatively identify penitrem A metabolites in blood plasma based on their accurate mass data and characteristic isotopic signature due to the presence of the chlorine atom. It has to be noted that penitrem A was more prominent in the extracted [M+H]^+^ ion chromatograms than any of the phase I metabolites. The peak height of metabolite M4_b relative to penitrem A was about 35%, similar to that of M1_c, which was 33%.

### 2.4. HRMS/MS Analysis of Putative Penitrem A Metabolites

**Figure 4:**
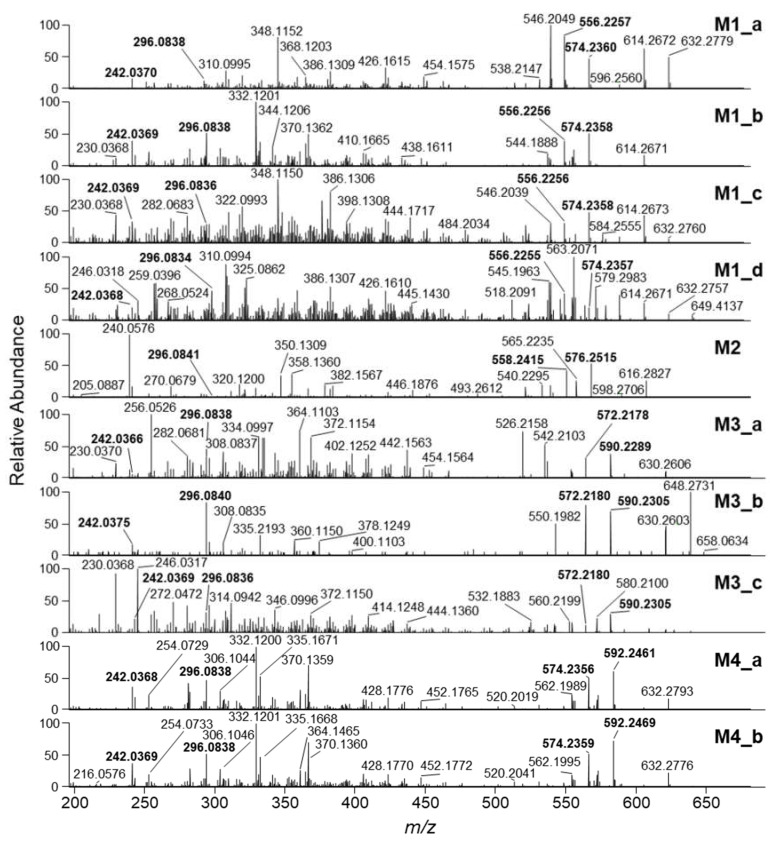
HRMS/MS product ion spectra from HCD of the [M+H]^+^ ions of putative penitrem A phase I metabolites produced in dog liver microsomes. Ions with *m*/*z* 242.037 and 296.084 were observed for all metabolites including penitrem A and could be suitable targets for penitrem A-specific fragment filtering. The bold-labeled higher-mass ions allowed distinguishing between the different metabolite types (i.e., *m*/*z* 556.226 and 574.236 for mono-oxygenated metabolites, *m*/*z* 558.242 and 576.252 for hydrated metabolites, *m*/*z* 572.218 and 592.229−592.231 for di-oxygenated metabolites, and *m*/*z* 574.236 and 592.246−592.247 for mono-oxygenated/hydrated metabolites). HRMS/MS spectra of good quality were not obtained for M1_e.

Parallel reaction monitoring targeting the [M+H]^+^ ions of putative penitrem A biotransformation products showed that they followed the same fragmentation pathways as the parent compound (Figure 4; Table 1). Thus, typical lower-mass product ions such as *m*/*z* 242.0368–242.0370 (C_14_H_9_NOCl^+^) and 296.0834–296.0841 (C_18_H_15_NOCl^+^), which were major fragments in the HRMS/MS spectra of penitrem A, were suitable for metabolite identification. They were used for fragment filtering of chromatograms from all-ion-fragmentation during the semi-targeted analysis of the plasma, tissue extracts and stomach contents of the four dogs that were suspected to suffer from penitrem poisoning. Moreover, several of the major higher- and medium-mass penitrem A product ions, for example, *m*/*z* 332.1187, 540.2282 and 558.2386, showed mass shifts in the product ion spectra of the metabolites that gave limited information about the molecular sites of the different biotransformation reactions. For example, the *m*/*z* 332.1187 product ion of penitrem A was observed with a mass shift corresponding to an oxygen atom in the spectra of M1_a and M1_c (i.e., *m*/*z* 348.1152 and 348.1150, respectively) (Figure 4), suggesting that the oxygenation had taken place in the substituted indole part of the molecule (Figure 2). In contrast, for M1_b, the product ion was observed at *m*/*z* 332.1201, indicating that the mono-oxygenation had not happened in the same moiety. The regioselectivity of the biotransformation reactions could, however, not be derived unambiguously from the MS/MS spectra because the dissociation energy required for breaking molecular bonds in the polycyclic penitrem A backbone is considerably higher than that needed to remove functional groups that were added by biotransformation reactions. Consequently, oxygen-containing functional groups (e.g. hydroxyl groups) will be lost during fragmentation before carbon–carbon bonds in the cyclic moieties of the molecular backbone are cleaved.

### 2.5. Kinetics of Penitrem A Depletion in Dog Liver Microsomes

**Figure 5:**
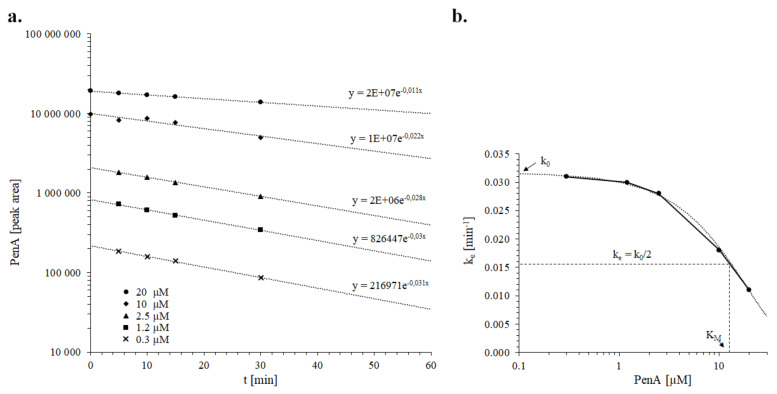
(**a**) Regression analysis for the determination of k_e_ at different penitrem A initial assay concentrations. (**b**) Approximation of K_M_ for penitrem A in incubations with dog liver microsomes. Regression of the concentration versus k_e_ curve allows convergence towards k_0_ at infinitesimally low substrate concentrations. The penitrem A concentration corresponding to k_0_/2 is equivalent to K_M_.

In vitro metabolism experiments with liver microsomes or primary hepatocytes can be used for the prediction of in vivo kinetic parameters if the assays are conducted under first-order kinetics [30,31]. Under the condition that the substrate concentration in the assay is well below the reaction constant (K_M,assay_), the assay clearance (CL_assay_) describing the elimination can be derived from the substrate depletion constant (k_e_). Therefore, as a first step, k_e_ for five different initial penitrem A assay concentrations were determined by regression analysis of concentration versus time curves (Figure 5a). Secondly, we approximated K_M,assay_ for penitrem A in incubations with dog liver microsomes as about 12 µM from the inflection point of a concentration versus k_e_ curve [32] (Figure 5b). All subsequent incubations were performed with starting concentrations of 1.2 or 2.5 μM penitrem A, thus fulfilling the requirements of linear kinetics.

### 2.6. Prediction of Toxicokinetic Parameters for Penitrem A in Dogs

The elimination half-life of penitrem A in the dog liver microsome assay (t_1/2,assay_) and the assay clearance (CL_assay_) were calculated from the k_e_ and used to determine the intrinsic assay clearance (CL_int,assay_), which gave a measure for the phase I enzyme activities in the microsomes (Table 3). The CL_int,assay_ was upscaled to the assay-independent, intrinsic liver clearance (CL_int_) by considering the microsomal protein amount and dog-specific upscaling factors for relative liver weight and microsomal recovery index [33,34]. IVIVE predicting the in vivo blood clearance (CL_b_) was performed by using the well-stirred liver model under consideration of the hepatic blood flow in dog [30]. With 0.9 (L/(h×kg)), the CL_b_ is considered intermediate to low, indicating that penitrem A is eliminated with medium velocity in dogs. Our prediction fitted thus with the in vivo-observed maximum toxic effect at about 30 min after intoxication [9]. Calculation of the maximal bioavailability (f_max_ = 57%) after oral application under the assumption of complete absorption from the gastrointestinal tract supported the assumption that a considerable amount of penitrem A reaches the systemic circulation after the first pass through the liver [35].

## 3. Conclusions

Penitrem A poisoning affects mostly dogs due to their feeding behavior and access to moldy food. Whereas the clinical symptoms of an intoxication are well-known, there are practically no data on the biotransformation and toxicokinetics of the toxin in dogs. In the present study, we have used HRMS to detect eleven penitrem A phase I-metabolites in incubations with dog liver microsomes. The identified metabolites included several isomers of mono- and di-oxygenated as well as hydrated products. They were confirmed in the plasma of a dog that had been exposed to penitrem A. Moreover, we applied in vitro-to-in vivo extrapolation of the substrate depletion data to predict important toxicokinetic parameters. The bioavailability of penitrem A is expected to be considerable and the blood clearance low enough to allow reaching a substantial toxin level in the systemic circulation.

## 4. Materials and Methods

### 4.1. Chemicals and Reagents

Crystalline penitrem A (≥95%), magnesium chloride hexahydrate, glucose-6-phosphate sodium salt, β-nicotinamide adenine dinucleotide phosphate sodium salt (NADP^+^), β-nicotinamide adenine dinucleotide phosphate reduced tetrasodium salt (NADPH), glucose-6-phosphate dehydrogenase, HEPES buffer and ammonium formate were from Sigma-Aldrich (Merck KGaA, Darmstadt, Germany). Water and acetonitrile (MeCN) for LC–MS were of Optima grade (Thermo Fisher Scientific, Waltham, MA, USA). Methanol (MeOH) and MeCN for other purposes than LC–MS were of gradient quality and from Romil (Cambridge, UK).

### 4.2. Microsomal Incubations

Substrate depletion assays measuring the concentration-time course of penitrem A were performed with commercially available dog liver microsomes (No. M00201, Lot #ORT, 20 mg/mL; Celsis, Baltimore, MD, USA) under conditions of first-order kinetics to determine assay half-life (t_1/2,assay_) for the extrapolation of toxicokinetic parameters. In vitro assays at higher concentrations were used to produce sufficient amounts of penitrem A metabolites for structure elucidation and identification.

From a penitrem A stock solution (0.5 mg/mL in MeOH, 790 μM), concentrated aliquots and dilutions were prepared in order to obtain final concentrations of 0.3, 1.2, 2.5, 10, and 20 μM in the microsomal assay. The proportion of organic solvent in the microsomal assay was less than 0.3%. The penitrem A solutions were added to 1 mL reaction mixture containing 2 mg/mL microsomes and an NADPH generating system (1 mM NADPH, 1 mM NADP^+^, 20.9 mM glucose 6-phosphate, 1 U/mL glucose-6-phosphate dehydrogenase, 9 mM MgCl_2_ × (H_2_O)_6_, 49 mM HEPES pH 7.4) after preincubation at 37 °C for 3 min in a shaking water bath (OLS 200; Grant, Cambridge, UK). The reaction tube was vortexed for 15 s and further incubated under shaking.

Aliquots of 130 μL were taken after 0, 5, 10, 15, 30 and 60 min and transferred to Eppendorf tubes containing equal volumes of ice-cold 100% MeCN. Samples were vortexed for 15 s and kept on ice until centrifugation at 20,000× *g* for 10 min at 4 °C to precipitate proteins (Eppendorf, Hamburg, Germany). Supernatants were filtered through 0.22 μm Nylon Costar Spin-X tubes (Corning Inc., Corning, NY, USA) by centrifugation at 15,000 × *g* for 1 min and transferred to chromatography vials for LC–ITMS analysis.

Penitrem A biotransformation products were concentrated prior to LC–HRMS analyses. Aliquots from the microsomal assay, taken between 10 to 60 min from incubations with 1.2 μM and 2.5 μM start concentrations were pooled (3.05 mL in total) in a conical tube. Most of the MeCN was evaporated at 50 °C under a nitrogen stream, and evaporation was stopped when the solvent level reached 1.8 mL. Ethyl acetate (1.8 mL; Rathburn Chemicals, Walkerburn, Scotland) was then added and vortexed for 1 min. After phase separation, the upper layer was carefully pipetted into a new tube. The ethyl acetate phase was evaporated to dryness, and the residue dissolved in 250 μL 80% MeOH and transferred to a HPLC vial.

### 4.3. Determination of Toxicokinetic Parameters In Vitro and Upscaling to In Vivo

The biotransformation assays were run under the conditions of first-order kinetics to allow the calculation of kinetic parameters for upscaling to in vivo. The activity of the microsomal enzymes can be described by the Michaelis–Menten equation parameters’ maximum velocity (v_max,assay_) and reaction constant (K_M,assay_) if the substrate concentration is well below the K_M_ value (CL_int,assay_ = v_max,assay_/K_M,assay_). As a first step, the K_M_ of penitrem A depletion in dog liver microsomes was approximated by using rate constants (k_e_) that were determined by regression analysis of measured peak areas versus time curves in experiments with different penitrem A start concentrations (0.3, 1.2, 2.5, 10 and 20 µM). The inflection point of a lin-log k_e_ versus concentration curve is equivalent to K_M_, occurring when k_e_ is half of the theoretical maximum k_0_ at infinitesimally low penitrem A concentrations [32]. The k_e_ of incubations with low starting concentration were subsequently used to calculate the assay half-life (t_1/2,assay_= ln2/k_e_) and, under consideration of the assay volume (V_assay_), the assay clearance (CL_assay_=V_assay_ × k_e_), which was close to the intrinsic assay clearance (CL_int,assay_), if protein binding of the substrate in the reaction mixture could be neglected.

The assay-independent intrinsic liver clearance (CL_int_=CL_int,assay_×MRI×RLW/Prot_assay_) for penitrem A in dogs was calculated under consideration of the amount of microsomal protein in the assay (Prot_Assay_), the dog-specific relative liver weight (RLW, 32 g/kg body weight) and the microsomal recovery index (MRI, 55 mg/g liver) [33,34]. The systemic blood clearance (CL_b_) was then determined by using the well-stirred liver model (CL_b_= Q × CL_int_/(Q + CL_int_) from the hepatic blood flow in dogs (Q, 2.1 L/(h×kg bodyweight) without consideration of binding to blood proteins [30,35]. The maximal bioavailability (f_max_) after oral application was calculated under the assumption of complete absorption from the gastrointestinal tract (f_max_=1–CL_b_/Q) [35].

### 4.4. LC–ITMS

Low-resolution mass spectrometry data were acquired using an LTQ linear ion trap mass spectrometer equipped with an atmospheric pressure chemical ionization (APCI) interface, and a Finnigan Surveyor MS Pump Plus and Accela autosampler (all Thermo Fisher Scientific). Aliquots from microsomal incubations were separated using a Kinetex XB-C18 column (100 × 4.6 mm, 2.6 μm particles; Phenomenex, Torrance, CA, USA) held at 30 °C with mobile phases A and B of water and 95% MeCN, respectively, both containing 2 mM ammonium formate, at a flow rate of 0.7 mL min^−1^. The column was eluted isocratically for 0.5 min with 30% B followed by a linear gradient increasing from 30% to 80% B in 17 min and finally to 95% B in 0.5 min. After flushing the column with 95% B for 3.5 min, the mobile phase was returned to 30% B in 0.5 min and re-equilibrated for 3.5 min. The mass spectrometer was operated in full scan ion mode (*m*/*z* 400–800), and the APCI interface in the positive ionization mode. The parameters for APCI were: vaporization temperature 400 °C, sheath gas flow 43 units nitrogen, auxiliary gas rate 7 units nitrogen, sweep gas flow rate 0 units, source voltage 6 kV, tube lens offset 90 V, capillary voltage 31 V and capillary temperature 200 °C. Toxicokinetic calculations were based on peak areas of the protonated molecules of penitrem A observed at *m/z* 634.2.

### 4.5. LC–HRMS and HRMS/MS

High-resolution mass spectrometry data with the aim to tentatively characterize penitrem A biotransformation products were collected using a Q-Exactive Quadrupole Orbitrap mass spectrometer equipped with a HESI-II heated electrospray ionization interface, and a Vanquish Horizon UHPLC pump, including an autosampler and column oven (all Thermo Fisher Scientific). Separation was achieved using a Kinetex EVO C18 column (100 × 2.1 mm, 2.6 µm; Phenomenex) held at 30 °C with mobile phases A and B of water and 95% MeCN, respectively, both containing 2 mM ammonium formate, at a flow rate of 0.3 mL min^−1^. The penitrem A metabolites were chromatographically resolved by isocratic elution for 0.5 min at 30% B followed by a linear gradient increasing from 30% to 80% B in 16.5 min and finally to 95% B in 0.5 min. After washing the column at 95% B for 3.5 min, the mobile phase was returned to 30% B in 0.5 min and re-equilibrated for 3.5 min. The total run time was 25 min. The conditions for positive HESI were as follows: capillary voltage, 3.5 kV; capillary temperature, 280 °C; sheath gas and auxiliary gas flow, 35 and 10 units, respectively; probe heater temperature, 300 °C; S-lens RF level, 55. The mass spectrometer was run in the Full-MS mode (scan range *m*/*z* 400–800) at a mass resolution set to 70,000 at *m*/*z* 200. The Full-MS scan was alternated with all-ion-fragmentation (AIF) of the ions in the same mass range, and product ions were scanned in the range *m*/*z* 80-800. The maximum target capacity of the C-trap (AGC target) was set to 3 × 10^6^ and the maximum injection time to 200 ms. Parallel reaction monitoring was performed using a mass resolution of 35,000 at *m*/*z* 200, an AGC target of 2 × 10^5^ and a maximum injection time of 100 ms. Precursor ions were selected with an isolation width of *m*/*z* 1.5 and fragmented using a normalized collision energy of 35 units. Product ions were scanned in the range *m*/*z* 50–735. Xcalibur (Thermo Fisher Scientific), version 2.3 was used for instrument control, while version 4.2 was used for data processing and calculation of elemental compositions.

### 4.6. Preparation of Stomach Contents and Plasma Samples from Potentially Intoxicated Dogs

Blood samples were obtained from four dogs suspected to suffer from acute penitrem A poisoning (Table 4). In addition, stomach contents were available from one of the dogs.

Stomach contents (ca. 13 g) were transferred to a 250 mL Beckmann centrifuge flask and 50 mL of 80% MeCN were added. The mixture was shaken for 20 min on an orbital shaker (250 min^−1^) (Edmund Bühler, Bodelshausen, Germany) and centrifuged in a Beckman J2-MC centrifuge (Beckman Coulter Life Sciences, Indianapolis, IN, USA) for 5 min at 2400 × *g*. An aliquot (500 μL) of the supernatant was filtered through 0.22 μm Nylon Costar Spin-X tubes (Costar, Corning Inc., Corning, NY, USA) at 20,000× *g* and 4 °C, transferred to an HPLC vial and submitted to LC–HRMS analysis.

Aliquots of blood plasma or serum (250 μL) were transferred to Eppendorf tubes containing 250 μL ice-cold 100% MeCN and vortexed for 30 s. After centrifugation at 20,000× *g* for 3 min at 4 °C (MicroCL 21R, Thermo Fisher Scientific), the supernatant was transferred to HPLC vials and submitted to LC–HRMS analysis.

### 4.7. Anamnesis of the Penitrem A-Positive Dog

Shortly after the owner had observed unusual behavior, he took the dog to the veterinary clinic at 10:30 pm. The dog had seizures, mydriasis, nystagmus and opisthotonos. Heparin plasma was taken for mycotoxin analysis, as well as other analyses, before medical treatment for seizures was started immediately. On clinical examination, the dog showed severe signs of depression, which made further neurological examinations difficult. Further indicators for a neurotoxic poisoning were posterior paresis and reduced or lacking withdrawal reflexes. The body temperature, heart rate and respiratory rate were normal. The only aberration found was a slight hyperglycemia. The dog alternated sleeping with tendencies of tremors and seizures during the night. At 10:00 am, the dog “awakened” and appeared more responsive, still having some tremors. Although still paretic and ataxic, there was a slight tendency of tonus in all four limbs. The dog was interested in feed and was able to swallow normally. It didn’t receive any medication and left the veterinary clinic the day after admission, making a full recovery.

## Figures and Tables

**Table 1:** HCD-generated main product ions of protonated penitrem A (^35^Cl isotopologue) including calculated elemental compositions, mass errors, ring double bond equivalents and suggestions for possible neutral losses.

*m/z*	Formula	Δ*m* (ppm)	RDBeq ^1^	Neutral Loss
616.2789	C_37_H_43_NO_5_Cl	−5.7	16.5	H_2_O
598.2694	C_37_H_41_NO_4_Cl	−4.1	17.5	2×H_2_O
564.2122	C_32_H_35_NO_6_Cl	−4.5	15.5	C_5_H_10_
558.2386	C_34_H_37_NO_4_Cl	−3.5	16.5	H_2_O + C_3_H_6_(O) ^2^
540.2282	C_34_H_35_NO_3_Cl	−3.2	17.5	2×H_2_O + C_3_H_6_(O) ^2^
528.1919	C_32_H_31_NO_4_Cl	−3.2	17.5	2×H_2_O + C_5_H_10_
428.1751	C_28_H_27_NOCl	−5.8	15.5	2×H_2_O + C_3_H_6_(O) ^2^ + C_6_H_8_O_2_
412.1440	C_27_H_23_NOCl	−5.5	16.5	2×H_2_O + C_3_H_6_(O) ^2^ + C_6_H_8_O_2_ + CH_4_
370.1341	C_25_H_21_NCl	−4.3	15.5	
332.1187	C_22_H_19_NCl	−4.1	13.5	
296.0826	C_18_H_15_NOCl	−3.6	11.5	
242.0357	C_14_H_9_NOCl	−4.2	10.5	

^1^ Ring double bond equivalents. ^2^ Acetone.

**Table 2:** LC–HRMS characteristics of putative biotransformation products of penitrem A in incubations with dog liver microsomes.

Metabolite ID	t_R_ (min) ^1^	*m/z*	Ion	Formula	Δ*m* (ppm)	RDBeq ^2^
Penitrem A	13.42	634.2927	[M+H]^+^	C_37_H_45_NO_6_Cl	−0.46	16
M1_a	8.81	650.2875	[M+H]^+^	C_37_H_45_NO_7_Cl	−0.58	16
M1_b	9.97	650.2876	[M+H]^+^	C_37_H_45_NO_7_Cl	−0.44	16
M1_c	10.09	650.2858	[M+H]^+^	C_37_H_45_NO_7_Cl	−3.5	16
M1_d	10.31	650.2865	[M+H]^+^	C_37_H_45_NO_7_Cl	−2.2	16
M1_e	10.87	650.2868	[M+H]^+^	C_37_H_45_NO_7_Cl	−1.6	16
M2	8.06	652.3046	[M+H]^+^	C_37_H_47_NO_7_Cl	1.6	15
M3_a	6.73	666.2834	[M+H]^+^	C_37_H_45_NO_8_Cl	0.81	16
M3_b	7.67	666.2831	[M+H]^+^	C_37_H_45_NO_8_Cl	0.34	16
M3_c	8.13	666.2826	[M+H]^+^	C_37_H_45_NO_8_Cl	−0.39	16
M4_a	9.02	668.2984	[M+H]^+^	C_37_H_47_NO_8_Cl	−0.17	15
M4_b	9.18	668.2987	[M+H]^+^	C_37_H_47_NO_8_Cl	0.28	15

^1^ t_R_: retention time, ^2^ ring double bond equivalents of the neutral molecule.

**Table 3:** Predicted toxicokinetic parameters for penitrem A in dogs by in vitro-to-in vivo extrapolation from incubations with dog liver microsomes.

Parameter	Dog Liver Microsomes
k_e_ (min^−1^)	0.03
t_1/2,assay_ (min)	23.1
K_M,assay_ (µM)	12
CL_int,assay_ (L/h)	1.8 × 10^−3^
CL_int_ (L/(h*kg))	1.6
CL_b_ (L/(h*kg))	0.9
f_max_ (%)	57

**Table 4:** Sample types and background information for four dogs with suspected penitrem poisoning.

Dog nr.	Sampling Date	Available Samples	Origin	Penitrem A
1	14 February 2018	Plasma, stomach contents	Fredrikstad ^1^ animal clinic	positive
2	25 February 2018	Plasma	Fredrikstad animal clinic	negative
3	22 January 2018	Serum	Fredrikstad animal clinic	negative
4	1 April 2018	Plasma	NMBU ^2^ small animal clinic	negative

^1^ Fredrikstad, Norway; ^2^ NMBU: Norwegian University of Life Sciences, Oslo, Norway.
